# Seizure outcomes and prognostic factors in patients with gangliogliomas associated with epilepsy

**DOI:** 10.3389/fsurg.2022.946201

**Published:** 2022-08-11

**Authors:** Yue Hu, Huawei Zhang, Aihemaitiniyazi Adilijiang, Jian Zhou, Yuguang Guan, Xueling Qi, Mengyang Wang, Jing Wang, Xiongfei Wang, Changqing Liu, Guoming Luan

**Affiliations:** ^1^ Department of Neurosurgery, Sanbo Brain Hospital, Capital Medical University, Beijing, China; ^2^ Beijing Institute for Brain Disorders, Capital Medical University, Beijing, China; ^3^ Department of Pathology, Sanbo Brain Hospital, Capital Medical University, Beijing, China; ^4^ Department of Neurology, Sanbo Brain Hospital, Capital Medical University, Beijing, China

**Keywords:** ganglioglioma, epilepsy, surgical resection, seizure outcome, prognostic factor

## Abstract

**Introduction:**

Ganglioglioma (GG) patients often present with seizures. Although most patients can be seizure-free after tumor resection, some still experience seizures. The present study aimed to analyze a group of GGs patients associated with epilepsy and evaluate the seizure outcomes and prognostic factors.

**Methods:**

This retrospective study involved clinical data collected from medical records of patients diagnosed with GG pathologically and underwent surgical resection in Sanbo Brain Hospital, Capital Medical University. The seizure outcomes were evaluated based on the International League Against Epilepsy (ILAE) seizure outcome classification. The prognostic factors were identified according to univariate and multivariate analysis.

**Results:**

A total of 222 patients were included, with a mean age at surgery of 19.19 ± 10.93 years. All patients were followed up at least for one year with a mean follow-up duration of 6.28 ± 3.17 years. At the final follow-up, 174 (78.4%) patients achieved ILAE Class 1 or 2. Univariate and multivariate analyses revealed that the short duration of seizures and gross total resection were significant positive factors for seizure-free. Bilateral interictal or ictal epileptiform discharges in preoperative video-electroencephalogram (VEEG) were related to poor seizure outcomes.

**Conclusion:**

Surgical resection is an effective treatment for patients with epilepsy associated with GGs. The analysis of predictive factors could effectively guide clinical practice and evaluate the prognosis of epilepsy with GG.

## Introduction

Epilepsy is one of the most common nervous system disorders, with an incidence of 6.38%–7.60% (1). In the epilepsy surgery series, tumors of the central nervous system (CNS) ranked as the second frequent category of pathology causing epilepsy in adults (after hippocampal sclerosis) or children (after focal cortical dysplasia) (2). Although GG is a rare tumor accounting for 0.4%–1.3% of all brain tumors, it is one of the most common causes of tumor-associated epilepsy (3–6). Previous studies revealed that gross total resection could be achieved in 58.2% to 93.8% of patients with GGs. The rate of seizure freedom ranged from 59.4% to 93.3% (5, 7–11).

Some clinical studies have reported that surgical resection can alleviate seizures in GG patients related to epilepsy (5, 12), but a few patients still experience seizures (13, 14). A previous clinical study demonstrated that patients benefit little from only reducing seizure frequency (15), and despite having undergone epilepsy surgery, the quality of life of patients with postoperative seizures was far worse than that of patients without seizures (16). Although some articles reported the risk factors for postoperative seizure outcomes, a portion of these articles focused only on specific populations, such as pediatric patients, or on specific sites of GG, such as those in the temporal lobe. In addition, some articles only reported GG as part of the research object. Besides, previous research results only had a certain value to guide clinical work, as the study sample sizes were limited. Herein, we reported a case series involving 222 patients to describe the clinical characteristics of GGs patients and evaluate the seizure outcomes and prognostic predictors. To the best of our knowledge, this is the largest cohort study in a single center.

## Materials and methods

### Patient selection

This single-institution analysis was approved by the local ethics committee. The inclusion criteria were as follows: (1) patients admitted to Sanbo Brain Hospital, Capital Medical University, Beijing, China, from May 2008 to January 2021, and (2) postoperative pathologies confirming the presence of GGs. The exclusion criteria were as follows: (1) no seizures as the clinic symptom, (2) a history of resection surgery in other hospitals, (3) clinical, neuroradiological, electrophysiological, and neuropathological data unavailable for review, (4) patients did not undergo surgical resection, and (5) patients were lost to follow-up. Patients' medical records were retrospectively reviewed for detailed demographic and clinical variables. The tumors were classified according to the World Health Organization (WHO) classification of CNS tumors of 2021 (17).

### Preoperative evaluation

The non-invasive tests on all patients included routine presurgical evaluations, such as seizure semiology, detailed history, neurological examination, brain magnetic resonance imaging (MRI), and long-term video-electroencephalogram (VEEG). MRI scans contained T1-, T2- and fluid-attenuated inversion recovery (FLAIR) images. The tumor site, calcification, and cystic changes were reviewed by the neuroradiologist. The electrodes were placed according to the standard 10–20 system with 64- or 128-channel long-term video-EEG monitoring. Interictal epileptiform discharges (IEDs) were termed “regional” when the IEDs only involved one lobe or contiguous lobes, “unilateral” when the IEDs arose from the ipsilateral hemisphere of the tumor, and “bilateral” when the IEDs were nonlateralized and involved both hemispheres. For the patients whose seizures could be recorded, the ictal discharge patterns were classified as regional, unilateral and bilateral as the IEDs. Electrophysiologists and neurologists worked together to identify the epileptogenic zone (EZ) based on the results of VEEG and semiology. The seizure type classification was based on the version of the International League Against Epilepsy (ILAE) 2017 (18).

After a series of detailed presurgical evaluations, the suitability for epilepsy surgery was decided by a multidisciplinary team consisting of neuroradiologists, electrophysiologists, neurologists, and neurosurgeons. Usually, if the patient's VEEG showed that the epileptic discharge was localized and consistent with the symptomatological and neuroimaging findings, the patient could proceed directly to the surgical stage. Otherwise, patients need to enter into the second stage of evaluation. Some special non-invasive tests would be performed such as magnetoencephalography (MEG), positron emission tomography-computed tomography (PET-CT). The epileptogenic focus can be identified by non-invasive examination in most patients with GG, but the preoperative evaluation of some patients showed that the range of the epileptogenic area was incompletely consistent with the tumor. For example, when the GG was located in the lateral temporal lobe, the patient's semiology and imaging may also indicate abnormalities in the ipsilateral hippocampus. In addition, some patients may have GGs that overlap with functional areas. Therefore, subdural grids or depth electrodes were implanted with the robotic stereotactic assistant to identify the EZ.

### Surgical procedure

The surgical goal was the gross total resection of the tumor without any complications. Intra-operative electrocorticography (ECoG) and other neuromonitoring facilities were performed to delineate the EZ and identify the functional areas. 45 The surgical type was defined as “gross total resection (GTR)” if no residual tumor tissue was found on postoperative MRI, “near-total resection” if more than 90% of the tumor was removed, and “subtotal resection (STR)” if less than 90% of the tumor was removed. Histopathological reports confirmed by the pathologist revealed that the tissues had a typical structure of GGs composed of neoplastic, mature ganglion cells in combination with neoplastic glial cells.

### Follow-up and seizure outcome

All patients who underwent epilepsy surgery were evaluated by the operating neurosurgeon in outpatients every three months postoperatively in the first year and yearly after that. A repeat MRI and scalp EEG were necessary to identify whether the tumor and EZ were completely resected together at the first re-examination of all patients. The seizure outcomes were recorded according to the ILAE seizure outcome classification (19), with favorable seizure outcomes defined as ILAE Class 1 and 2 and unfavorable seizure outcomes defined as ILAE 3–6. All patients continued to take AEDs for prophylaxis after surgery. Whether to wean off AEDs or reduce the dosages of AEDs after surgery depended on the patients' seizure outcomes and EEG results. Patients taking monotherapy postoperatively would be able to wean off AEDs gradually if they met the following requirements: (1) no seizures for 2 years postoperatively, (2) no interictal epileptiform discharges on postoperative EEG, (3) no tumor recurrence on MRI. Patients who were on polytherapy postoperatively and met the above requirements could gradually reduce the type or dose of AEDs. Otherwise, AEDs treatment should be adjusted according to the patients' examination results.

### Statistical analysis

For continuous variables, means, standard deviation (SD), and ranges are presented. The continuous variables were stratified based on the Youden index in operating receiver curve (ROC) analysis to identify the threshold that might affect the seizure outcomes. Frequencies and percentages are presented for categorical data. The categorical data were assessed using Pearson X^2^ test or Fisher's z-test. Univariate and multivariate analyses were performed to identify the predictors of seizure outcomes. All statistical analyses were performed using SPSS statistics software version 25 (IBM). A *P*-value <0.05 indicated statistical significance. Kaplan-Meier analysis was used to calculate the cumulative rate of seizure-free and plot the survival curves.

## Results

### Demographic characteristics

**Figure 1:**
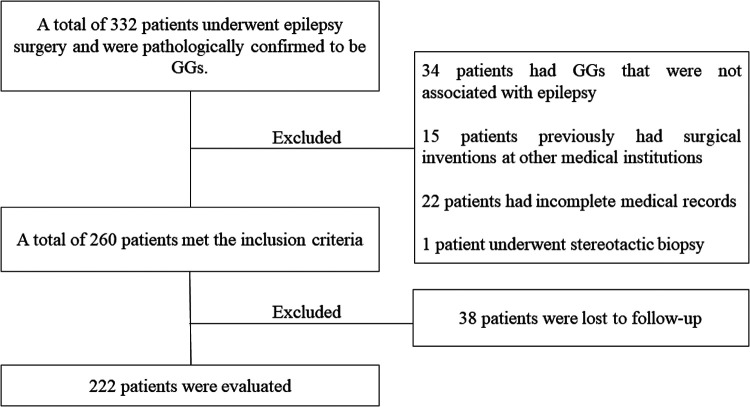
The patient-selection process.

Between May 2008 and January 2021, 222 patients (137 males and 85 females) met the inclusion criteria and were enrolled in this study. The mean age at the time of surgery was 19.19 ± 10.93 (range, 1.0–64.0) years, the mean age at seizure onset was 12.02 ± 9.74 (range, 0.0–63.9) years, and the mean duration of the seizures was 7.17 ± 7.38 (range, 0.1–32.9) years. The patient-selection process is displayed in Figure 1.

### Clinical characteristics

The clinical characteristics of all patients are presented in Table 1, showing that 95 (43.0%) patients had an epileptic aura before the seizures. The seizure frequencies were recorded daily in 73 (32.9%), weekly in 67 (30.2%), monthly in 63 (28.4%), and yearly in 19 (8.6%) patients. In the cohort, 61 (27.5%) had only focal-onset seizures, 58 (26.1%) patients had only generalized-onset seizures, and the remaining 103 (46.4%) patients had both seizure types. Seventeen patients (7.7%) did not take AEDs preoperatively, possibly due to the short duration or the low frequency of seizures, 33 (14.9%) patients were treated with monotherapy and 172 (77.4%) patients were treated by polytherapy. In the last follow-up, 76 of the 222 (34.2%) patients weaned off AEDs, 77 (34.7%) patients received monotherapy, and the remaining 69 (31.1%) patients were still receiving polytherapy. The mean number of AED after surgery (1.11 ± 1.07) was much lower than at baseline (2.01 ± 0.98) (*P* < 0.001).

**Table 1:** Demographic and clinic characteristics of patients and the relationship with seizure outcomes.

Characteristics	Favorable outcomes	Unfavorable outcomes	*P*
Sex			
M, *n* = 137	109 (79.6%)	28 (20.4%)	0.587
F, *n* = 85	65 (76.5%)	20 (23.5%)	
Age at seizure onset			
≤6 y, *n* = 86	67 (77.9%)	19 (22.1%)	0.892
>6 y, *n* = 136	107 (78.7%)	29 (21.3%)	
Duration of seizures			
≤2 y, *n* = 78	70 (89.7%)	8 (10.3%)	0.002*
>2 y, *n* = 144	104 (72.2%)	40 (27.8%)	
Age at surgery			
≤18 y, *n* = 96	79 (82.3%)	17 (17.7%)	0.216
>18 y, *n* = 126	95 (75.4%)	31 (24.6%)	
Aura			
Yes, *n* = 95	70 (73.7%)	25 (26.3%)	0.187
No, *n* = 127	104 (81.9%)	23 (18.1%)	
Seizure frequency			
Daily, *n* = 73	61 (83.6%)	12 (16.4%)	0.156
Weekly, *n* = 67	47 (70.1%)	20 (29.9%)	
Monthly, *n* = 63	49 (77.8%)	14 (22.2%)	
Yearly, *n* = 19	17 (89.5%)	2 (10.5%)	
History of SE			
Yes, *n* = 10	8 (80.0%)	2 (20.0%)	0.899
No, *n* = 212	166 (78.3%)	46 (21.7%)	
Seizure types			
Focal only, *n* = 61	52 (85.2%)	9 (14.8%)	0.296
Generalized only, *n* = 58	43 (74.1%)	15 (25.9%)	
Both, *n* = 103	79 (76.7%)	24 (23.3%)	
IEDs			
Regional, *n* = 82	71 (86.6%)	11 (13.4%)	0.003*
Unilateral, *n* = 48	38 (79.2%)	10 (20.8%)	
Bilateral, *n* = 53	32 (60.4%)	21 (39.6%)	
Nonspecific, *n* = 39	33 (84.6%)	6 (15.4%)	
Ictal onset rhythms			
Regional, *n* = 36	30 (83.3%)	6 (16.7%)	0.001*
Unilateral, *n* = 35	31 (88.6%)	4 (11.4%)	
Bilateral, *n* = 72	45 (62.5%)	27 (37.5%)	
Not captured, *n* = 79	68 (86.1%)	11 (13.9%)	
Surgical type^a^			
GTR, *n* = 140	118 (84.3%)	22 (15.7%)	<0.001*
Near-total resection, *n* = 65	50 (76.9%)	15 (23.1%)	
STR, *n* = 17	6 (35.3%)	11 (64.7%)	
Laterality of tumor in preoperative MRI			
Left, *n* = 110	88 (80.0%)	22 (20.0%)	0.561
Right, *n* = 112	86 (76.8%)	26 (23.2%)	
Classification of tumors^b^			
Low grade, *n* = 218	172 (78.9%)	46 (21.1%)	0.164
Anaplastic, *n* = 4	2 (50.00%)	2 (50.00%)	
Site of lesion			
Temporal, *n* = 173	138 (79.8%)	35 (20.2%)	0.344
Extratemporal, *n* = 49	36 (73.5%)	13 (26.5%)	
Acute postoperative seizures^c^			
Yes, *n* = 34	23 (67.6%)	11 (32.4%)	0.099
No, *n* = 188	151 (80.3%)	37 (19.7%)	

Abbreviations: M, male; F, female; SE, status epilepticus; MRI, magnetic resonance imaging; IED, interictal epileptic discharge; GTR, gross total resection; STR, subtotal resection.

^a^ Based on surgical records and postoperative neuroimaging.

^b^ Based on histopathological examination.

^c^ Seizures occurred during the first week after surgery.

* *P* < 0.05.

**Figure 2:**
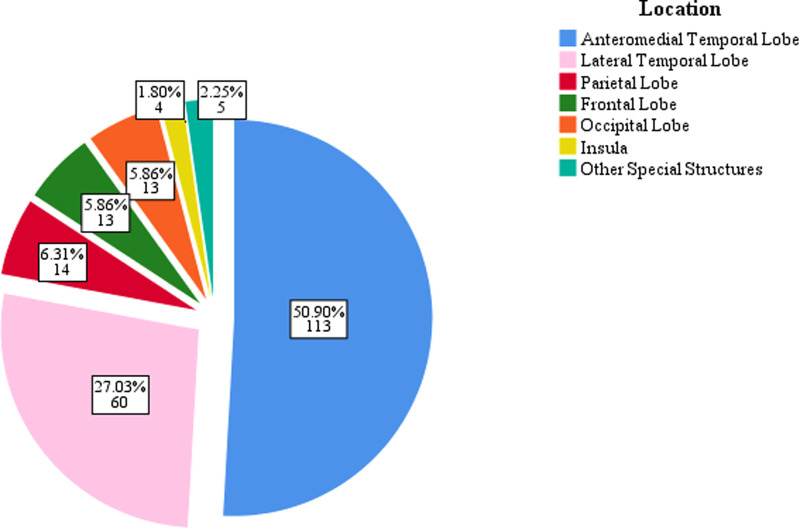
The location distribution of GG.

All patients underwent preoperative MRI examination. Tumor located in temporal lobe were found in 173 patients (77.9%), of which 113 (50.9%) were located in the anteromedial temporal lobe, and 60 (27.0%) were located in the lateral temporal lobe. Tumor located in the parietal lobe, frontal lobe, and occipital lobe in 14 (6.3%), 13 (5.9%), and 13 (5.9%) patients, respectively (Figure 2). Scalp EEG monitoring results were obtained for all patients. IEDs were regional in 82 (36.9%) patients, unilateral in 48 (21.6%), and bilateral in 53 (23.9%) patients, with nonspecific findings in 39 (17.6%) patients. Ictal onset rhythms were regional in 36 (16.2%), unilateral in 35 (15.8%) and bilateral in 72 (32.4%) patients. The EEG monitoring time was insufficient, so the seizure could not be captured in 79 (35.59%) patients. For accurate localization of epileptic foci, 95 (42.8%) patients underwent MEG, 49 (22.1%) underwent PET-CT, and 14 (6.3%) underwent intracranial electrode implantation.

GTR of the tumor was achieved in 140 (63.1%) cases, near-total resection was achieved in 65 (29.3), and STR was achieved in 17 (7.7%) patients. Histopathological reports revealed that the tissues had a typical structure of GGs composed of neoplastic, mature ganglion cells in combination with neoplastic glial cells. A total of 218 (98.2%) were classified as low-grade tumors, and 4 (1.8%) were anaplastic according to the WHO classification of CNS tumors. Subsequently, the neocortex surrounding the tumor was characterized by cortical disorganization in 98 (44.1%) cases, of which 38 (17.1%) had typical focal cortical dysplasia (FCD). Among the 38 patients with FCD, 3 with FCD Ia, 18 with FCD Ib, 9 with FCD IIb, and 8 with FCD IIIb.

### Surgical complications

In this case series, two patients died of tumor recurrence at the last follow-up. A total of 35 (15.8%) patients had transient neurological deficits or complications that did not affect their quality of life, including 7 (3.2%) with muscle weakness, 9 (4.1%) with contralateral quarter-quadrant hemianopia, 7 (3.2%) with memory impairment, 3 (1.4%) with transient dysphasia, 3 (1.4%) with intracranial infection, 2 (0.9%) with intracranial hematoma, 1 (0.5%) with wound infection, and 3 (1.4%) with cerebrospinal fluid leakage. All 35 patients returned to work or study after comprehensive treatment or rehabilitation. In addition, 25 (11.3%) patients suffered permanent neurological deficits, 13 (5.9%) had hemiparesis, 4 (1.8%) had facial paresis, 4 (1.8%) had dysphasia, including 1 (0.5%) patient with motor aphasia and 3 (1.4%) patients with sensory aphasia, 3 (1.4%) had hemianopia, and 1 (0.5%) had paresthesia. Although the patients were treated by postoperative rehabilitation training, they still had symptoms, which affected their lives. It should be noted that the dysfunction existing preoperatively was not included in the surgical complications.

### Follow-up and outcomes

Six patients underwent reoperation. Two of them underwent hematoma removal due to postoperative CT showing intracranial hematoma. Four patients had tumors that overlapped with functional areas, and preoperatively they could not accept the complications that might affect their quality of life. However, the postoperative seizure outcomes were unfavorable, and they underwent reoperation. Ultimately, these four patients achieved seizure-free, but one patient had sensory aphasia and three patients had hemiparesis. All patients were followed up for at least one year, with a mean follow-up duration of 6.28 ± 3.17 (range, 1.01–13.76). At the last follow-up, 174 (78.4%) patients achieved favorable seizure outcomes. Among the 222 patients, 12 had seizures only once due to missing antiepileptic drugs, so these patients were also classified into the group who achieved favorable outcomes.

### Prognostic factors

**Figure 3:**
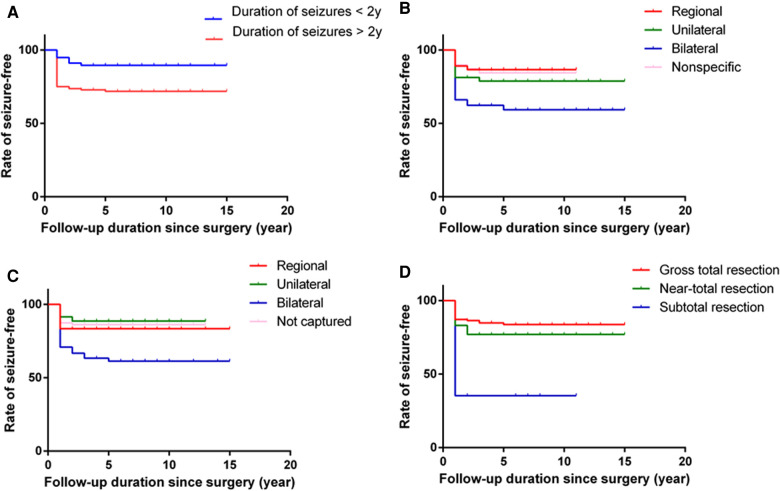
Kaplan-Meier curves for seizure freedom. Graphs demonstrate cumulative rate of seizure free over the follow-up period after epilepsy surgery for GGs by duration of seizures (**A**), IEDs (**B**), ictal onset rhythms (**C**) and surgical types (**D**).

In the univariate analysis, the potential prognostic factors associated with seizure outcomes were as follows: duration of seizures (*P* = 0.002), IEDs (*P* = 0.003), ictal onset rhythm (*P* = 0.001), surgical type (*P* < 0.001). The other factors which may not affect the seizure outcomes are listed in Table 1. A binary logistic regression model in a backward fashion was applied to evaluate the above-mentioned factors further, revealing that the duration of seizures > two years (odds ratio (OR) = 3.980, confidence interval (CI: 1.544–10.256, *P* = 0.004), bilateral IEDs (OR = 3.134, CI: 1.178–8.337, *P* = 0.022), bilateral ictal onset rhythms (OR = 3.630, CI: 1.088–12.112, *P* = 0.036) and PR (OR=22.040, CI: 4.788–101.465, *P* < 0.001) were associated with unfavorable seizure outcomes (Table 2). The Kaplan-Meier analyses for the significant factors are described in Figure 3.

**Table 2:** The potential prognostic factors associated with seizure outcomes on multivariate analysis.

Characteristics	OR	(95% CI)	*P*
Duration of seizures >2 y	3.980	1.544–10.256	0.004^*^
Bilateral IEDs	3.134	1.178–8.337	0.022^*^
Bilateral Ictal onset rhythms	3.630	1.088–12.112	0.036^*^
Subtotal resection	22.040	4.788–101.465	<0.001^*^

Abbreviations: OR, odds ratio; CI, confidence interval.

* *P* < 0.05.

## Discussion

Seizures are the common manifestations of GGs, and despite low incidence, GG comprises approximately 40% of long-term tumor-associated epilepsy (10, 16). It has been demonstrated that GGs primarily occur in children and young adults and more commonly involve the temporal lobe (3, 20–22). Although previous studies have reported these characteristics of GGs, only a few studies were conducted with small sample sizes to analyze the prognostic factors of epilepsy associated with GG. The present study involved 222 patients with epilepsy secondary to GG who underwent surgical resection. To our knowledge, this is the largest case series on GG.

**Figure 4:**
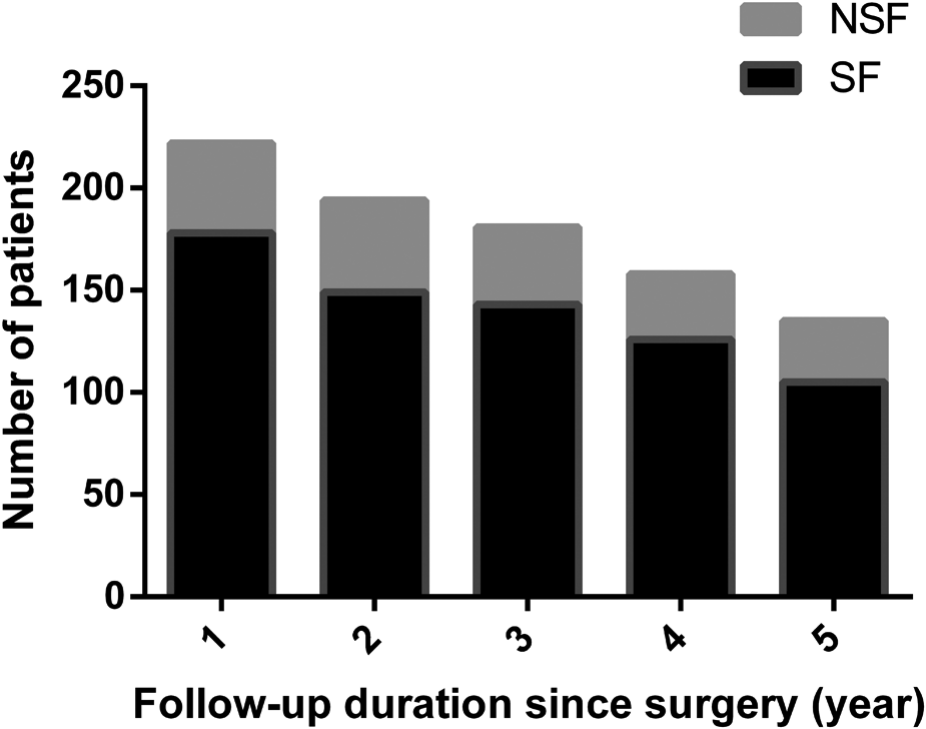
Seizure outcomes according to follow-up duration since surgery.

In this retrospective study, the mean follow-up time was 6.28 ± 3.17 (range, 1.01–13.76) years, and most (78.38%) patients were seizure-free, similar to previous studies reporting a rate of 59.4%–93.3% (7, 8, 11, 13, 23–25). Southwell et al. found that the excellent seizure outcomes were stable, with 80% of patients being seizure-free ten years post-surgery (9). In our case series, long-term surgical outcomes could be obtained in 135 patients who were followed up for more than five years, 105 remained ILAE Class 1 or 2, and the seizure-free rate reached 77.8%. There was no significant difference between short-term results and long-term results in the present study (Figure 4).

Studies focusing on patients with GG have identified some factors associated with favorable seizure outcomes, such as age at surgery, duration of epilepsy, completeness of tumor, etc. (8, 10, 13, 26–28). In this clinical cohort study, the duration of seizures > two years was a potential factor associated with unfavorable seizure outcomes. Huang et al. also demonstrated that patients with a short duration of seizures were more likely to be seizure-free following resection (10). Other studies reported a significant benefit of both shorter duration of seizures and younger age at surgery (9, 29). However, it could not be concluded that younger age at surgery helped to improve the rate of seizure freedom in the present study. The exact reason for the phenomenon was unknown, but the long-term existence of the tumor and seizures may cause structural damage in the peritumoral cortex. Since recurrent seizures would lead to cognitive dysfunction and seriously affect the quality of life of patients (14, 30), early surgical treatment for patients, especially children with epilepsy secondary to GGs, is recommended.

Lee et al. and Mittal et al. found that bilateral epileptiform discharges were associated with unfavorable seizure outcomes in patients with brain tumors (31, 32), similar to the present study. This phenomenon could be attributed to the fact that epileptic discharges rapidly spread to the contralateral hemisphere in the early stage, or recurrent seizures result in secondary epileptogenic foci. If the surgeon only focused on tumor removal, the possibility of poor postoperative seizure control would increase. Furthermore, when the tumor is deep in the brain, the scalp EEG does not precisely locate the EZ. Previous studies demonstrated that MEG, PET-CT, and stereoelectroencephalography (SEEG) accurately identified the EZ (33–35). However, not all patients underwent these tests in the present study. Therefore, the association between these tests and seizure outcomes could not be evaluated.

Due to the low malignant potential, the survival rate was excellent for GG patients. Thus, not only tumor removal but also seizure control should be the surgical goal for treating GGs patients (3, 10, 13, 23). However, the extent of surgical resection in GG patients associated with epilepsy is still controversial (13, 14, 23, 36). In the present study, the seizure freedom rate was highest in patients who underwent GTR. Although the seizure outcome in near-total resection was not as good as in the former group, the difference was insignificant. In contrast, 17 patients underwent STR, and only 6 had a favorable outcome. Southwell et al. reported that in their study of 49 patients with GG associated with epilepsy, 14 underwent extended resection, and 22 underwent lesionectomy, with 13 and 21 patients, respectively, achieving good seizure outcomes. In comparison, 13 patients underwent PR, with only 7 having a good seizure outcome. In the study by Ogiwara et al. an excellent seizure outcome was also observed in patients who underwent GTR (37), whereas Hu et al. found no difference between GTR and STR of the tumor in postoperative seizure outcome (26). In addition to the short duration of epilepsy and complete tumor resection, some other factors may affect the epilepsy results of GG patients. Ozlen et al. analyzed the clinical results of 52 patients with dysembryoplastic neuroepithelial tumors and gangliogliomas (38), reporting that absence of status epilepticus (SE) and seizures within the first month of surgery was associated with better seizure outcomes. Although ten patients in this study had a history of status epilepticus, it was not associated with the seizure outcomes. Status epilepticus is a neurological emergency with high mortality (39), with over half the SE cases presenting for the first time without an existing diagnosis of epilepsy. Accurate identification and control of seizure could be beneficial to patient prognosis. Moreover, in this study, 34 patients had acute postoperative seizures, of which 11 had unfavorable seizure outcomes (*P* = 0.099). However, the causes of acute postoperative seizures are diverse, such as cortical irritation, hemorrhage, brain edema, and incomplete resection of epileptic focus (40, 41).

Although GGs had a predilection for temporal lobes, there was no significant difference in seizure outcomes between temporal vs. extratemporal tumors in this study. Cortical dysplasia could be peritumoral, which might be responsible for the recurrence of epilepsy (21, 23, 42). Interestingly, cortical disorganization was detected in the tissue adjacent to the tumor in 98 (44.14%) patients, of which 38 cases had typical FCD characteristics, indicating that cortical abnormalities were the structural basis of seizure recurrence in some patients after epilepsy surgery. Surgical resection of the tumor and the abnormal peritumoral cortex may increase the seizure-free rate. We reviewed the specific pathological types of these patients, showing that there were 3 with FCD Ia, 18 with FCD Ib, 9 with FCD IIb, and 8 with FCD IIIb. The *ad hoc* Task Force of ILAE released a new category of FCDs in 2011 (43). FCD type IIIb is the association of FCD type I with a glial or glioneuronal tumor. According to the new classification, FCD types of these 38 patients should include 9 FCD type II with GG and 29 FCD type III, of which 5 and 23 patients with these two pathological types, respectively, were seizure-free at the most recent follow-up (*P* = 0.205).

ECoG is routinely performed in epilepsy surgery in many centers to delineate the extent of the extratumoral EZ (24, 32). In our experience, EZ was delineated by ECoG before tumor resection, and after tumor removal, ECoG was applied again to confirm whether there were residual epileptogenic foci. If an active spike focus was found in the adjacent tissue, additional resection of those areas would be performed. Thus, the actual extent of resection was larger than the lesion volume in some patients. However, there are some limitations, including susceptibility to anesthesia disturbance, absence of ictal information, and short duration of monitoring. Mittal et al. performed a two-stage surgical approach to investigate the spatial relationship between the tumors and EZ (32) finding that the EZ could be inside or outside the tumors. Based on the perspective of seizure control, some investigators speculated that extensive resection might benefit the patients (23, 44, 45). However, most patients in this study only underwent a single-stage operation. Klink et al. considered that applying high-frequency oscillations in ECoG instead of epileptic spikes would improve seizure freedom rates in tumor-related epilepsy surgery (46). Nonetheless, there was no detailed data about intraoperative high-frequency oscillations in the review of the medical records.

### Limitations

Although the sample size was sufficiently large, it was collected from a single center. Moreover, it was a retrospective study, so methodological limitations could be unavoidable. Some other factors that contributed to seizure outcomes had not been considered, such as BRAF V600E gene mutation. Randomized controlled trials need to be performed regarding the effect of ECoG. GGs mostly originate from the temporal lobe. However, we did not further analyze the potential factors affecting cognitive function which significantly impacted the selection of treatment by either surgeons or patients. Substantial research is required to address this phenomenon in the future.

## Conclusions

This single-center retrospective cohort study revealed that most patients with GGs have seizures as the main clinical symptom, which can be resolved in most patients by surgical resection. GTR and a shorter duration of seizures are associated with a high probability of being seizure-free, whereas bilateral IEDs and ictal onset rhythms may be associated with unfavorable seizure outcomes. These characteristics are helpful for clinicians in predicting the prognosis of epilepsy associated with GG.

## Data Availability

The original contributions presented in the study are included in the article/Supplementary Material, further inquiries can be directed to the corresponding author/s.
